# Use of a dual lumen cannula for venovenous extra corporeal membrane oxygenation in a patient with acute respiratory distress syndrome and a previously inserted inferior vena cava filter: a case report

**DOI:** 10.5935/0103-507X.20160001

**Published:** 2016

**Authors:** Fernando Palizas Jr., Christian Casabella García, Mariano Norese

**Affiliations:** 1 Clínica Bazterrica - Buenos Aires, Argentina.

**Keywords:** Extracorporeal membrane oxygenation, Respiratory distress syndrome, adult, Inferior vena cava filter, Thoracic injury, Vena cava filters, Venous thrombosis, Case reports

## Abstract

Extracorporeal membrane oxygenation is used in refractory hypoxemia in many clinical settings. Thoracic trauma patients usually develop acute respiratory distress syndrome. Due to high risk of bleeding, thrombotic complications present in this context are particularly difficult to manage and usually require insertion of an inferior vena cava filter to prevent embolism from the distal veins to the pulmonary circulation. Here, we present a case of a thoracic trauma patient with severe acute respiratory distress syndrome requiring venovenous extracorporeal membrane oxygenation via a right internal jugular double lumen cannula due to a previously inserted inferior vena cava filter caused by distal bilateral calf muscle vein deep vein thrombosis.

## Abstract (pt)

A oxigenação por membrana extracorpórea é utilizada em casos de hipoxemia refratária em diversas condições clínicas. Pacientes vítimas de traumatismo torácico geralmente desenvolvem síndrome da angústia respiratória aguda. Em razão do elevado risco de sangramentos, as complicações trombóticas que se apresentam neste contexto são particularmente difíceis de tratar e, geralmente, demandam a inserção de um filtro na veia cava inferior, com a finalidade de prevenir a migração de êmbolos oriundos das veias distais para a circulação pulmonar. Neste artigo, apresentamos o caso de um paciente com traumatismo torácico, que apresentou grave síndrome de angústia respiratória aguda, com necessidade de utilizar oxigenação por membrana extracorpórea aplicada por meio da introdução de uma cânula com duplo-lúmen na veia jugular interna direita. Este procedimento foi realizado tendo em vista a prévia inserção de um filtro na veia cava inferior, por conta da ocorrência de trombose venosa profunda em ambas as panturrilhas.

## INTRODUCTION

Extracorporeal membrane oxygenation (ECMO) has been increasingly used to support patients with refractory hypoxemia. To provide adequate blood flow for oxygenation, the cannulation site and cannula size must be carefully selected. In acute respiratory distress syndrome (ARDS) patients, the venovenous mode is preferred, in which the femoro-jugular and right internal jugular (with double lumen cannulas) cannulation strategy is the most commonly used.^(1)^ Anticoagulation is usually required during the ECMO run to prevent thrombotic complications in the extracorporeal circuit and patient.

Trauma patients usually have bleeding and/or thrombotic complications and require multiple surgical procedures,^(2)^ and anticoagulation in this context is not always safe or even possible. We present a case of a blunt thoracic trauma patient complicated with severe ARDS placed on venovenous-ECMO with a dual lumen (DL) cannula via right internal jugular due to the presence of an inferior vena cava filter.

## CASE REPORT

A 68-year-old man with a prior history of smoking (30 pack/year) without chronic respiratory symptoms was admitted to our intensive care unit due to blunt thoracic trauma after a car accident. A computed tomography scan revealed a grade I left-side anterior pneumothorax, left lower lung contusion and 4 left rib fractures (rib number 1 to 4). Three ribs were fractured in two places. A complete examination revealed left scapulae fracture and mild traumatic brain injury without neurological symptoms and a normal computed tomography scan. His injury severity score was 18.

During the first day, the patient developed signs of respiratory insufficiency requiring supplemental oxygen via a Venturi mask, and eventually, non-invasive ventilation was started due to hypoxemia and chest wall paradoxical motion. Due to the progression of pulmonary infiltrates and worsening hypoxemia, he was intubated and placed on mechanical ventilation with a protective ventilatory strategy, and a left-side chest tube was inserted to drain the pneumothorax. Distal bilateral calf muscle vein thrombosis was diagnosed by ultrasound, and anticoagulation with enoxaparin was started. Antibiotics were started because the patient was febrile, and a Methicillin-sensitive *Staphylococcus Aureus* was recovered from a tracheal aspirate. On the following day, the patient developed a left-side hemothorax, which was drained using a chest tube, and the anticoagulation was stopped, and a retrievable inferior vena cava filter was implanted.

Two days later, the patient condition deteriorated; the patient developed ARDS (mean PaO_2_/FiO_2_ 160mmHG) and hemodynamic instability requiring vasopressors. Ventilator-associated pneumonia was diagnosed, and *Klebsiella Pneumoniae* was recovered from a bronchoalveolar lavage.

On the 7^th^ day, venovenous-ECMO was indicated because of severe ARDS with a PaO_2_/FiO_2_ ratio of 90 despite the use of lung protective ventilation and neuromuscular blockers. Prone positioning previous to ECMO was not considered due to left hemi-thorax instability and inhaled nitric oxide was not available in our institution.

**Figure 1:**
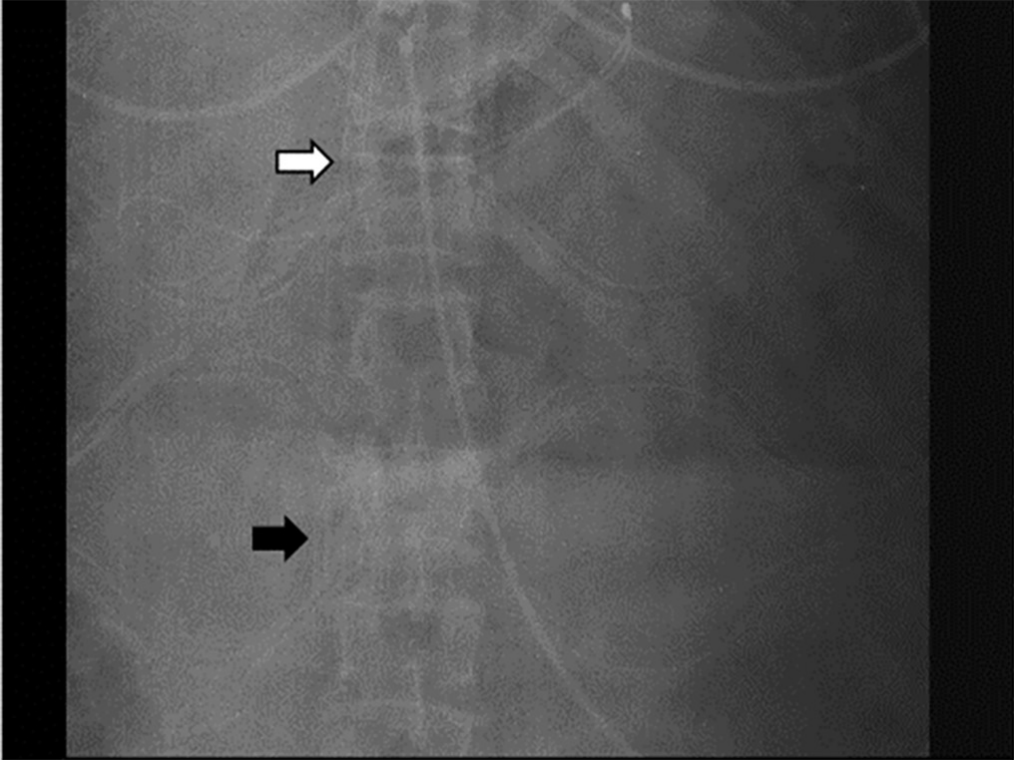
Double lumen cannula and inferior vena cava filter. Inferior vena cava filter and tip of the double lumen cannula was confirmed with daily x-rays due to potential filter migration. The white arrow indicates the tip of the double lumen cannula, and the black arrow indicates the inferior vena cava filter.

**Figure 2:**
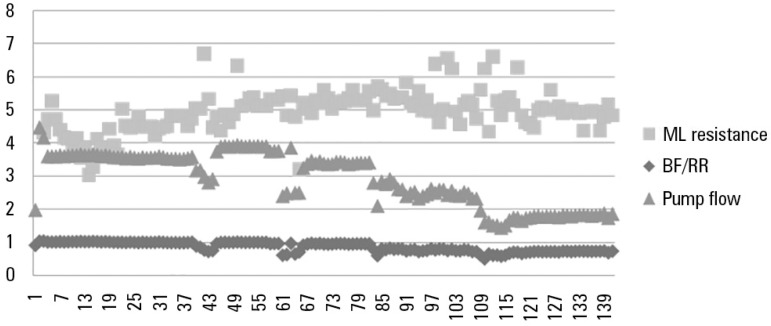
Extracorporeal membrane oxygenation circuit monitoring the entire run. There were no problems during the run with resistance to blood flow or with the pump load conditions, which was evaluated as blood flow to the rotation ratio. ML resistance - membrane lung resistance to blood flow; BF/RR - blood flow to rotation ratio.

A DL 27 French Avalon^®^ (Maquet Cardiopulmonary AG, Rastatt, Germany) right internal jugular cannula was placed percutaneously under fluoroscopic and transesophageal echocardiography control with a previous right internal jugular ultrasound showing the absence of thrombi. Because there were some concerns about inferior vena cava filter migration due to the suction generated by the ECMO pump, daily abdominal and chest x-rays were performed, and the distance between the inferior vena cava filter and the tip of the DL cannula remained unchanged during the entire ECMO run (Figure 1). No problems were detected related to the presence of the inferior vena cava filter and ECMO system. The ECMO flow, resistance of the oxygenator and blood flow to the rotation of the pump ratio, and system pressures remained within acceptable ranges (Figure 2). Recirculation was not measured; however, inlet venous saturation remained below 72%, suggesting little or no recirculation.

Respiratory rest settings were selected (Table 1). Sweep gas was adjusted to achieve an arterial CO_2_ of 40mmHg. Initial ECMO blood flow was set at 4L/min and then adjusted to achieve arterial oxygen saturation between 88 and 95%.

**Table 1:** Respiratory and organ function parameters before and after extracorporeal membrane oxygenation

	Pre ECMO	On ECMO
Respiratory rate (bpm)	22	10
Tidal volume (mL/Kg)	6.2	5
Pplat (cmH_2_O)	29	25
PEEP (cmH_2_O)	12	10
Driving pressure (cmH_2_O)	17	15
FiO_2_ (%)	90	30
SaO_2_ (%)	90	96
Cestrs (mL/cmH_2_O)	25.8	26
Respiratory minute volume (L/m)	9.7	3.9
SOFA	9	1
Noradrenaline (mcg/Kg/min)	0.2	0

ECMO - extracorporeal membrane oxygenation; Pplat - inspiratory plateau pressure; PEEP - positive end-expiratory pressure; FiO_2_ - fraction of inspired oxygen; SaO_2_ - arterial oxygen saturation; Cestrs - static compliance of the respiratory system; SOFA - Sequential Organ Failure Assessment.

The ECMO run lasted 140 hours without bleeding or thrombotic complications. No anticoagulation was used during the first 24 hours. Heparin was subsequently added in increasing doses (mean 22 units per kilogram per hour), and full anticoagulation (mean activated partial thromboplastin time: 66") was achieved by ECMO on the 35^th^ hour.

Oxygenation improved, and after a full day of weaning from ECMO, the patient was percutaneously decannulated. Few fibrin deposits were observed on the venous side of the oxygenator without significantly affecting membrane function.

**Figure 3:**
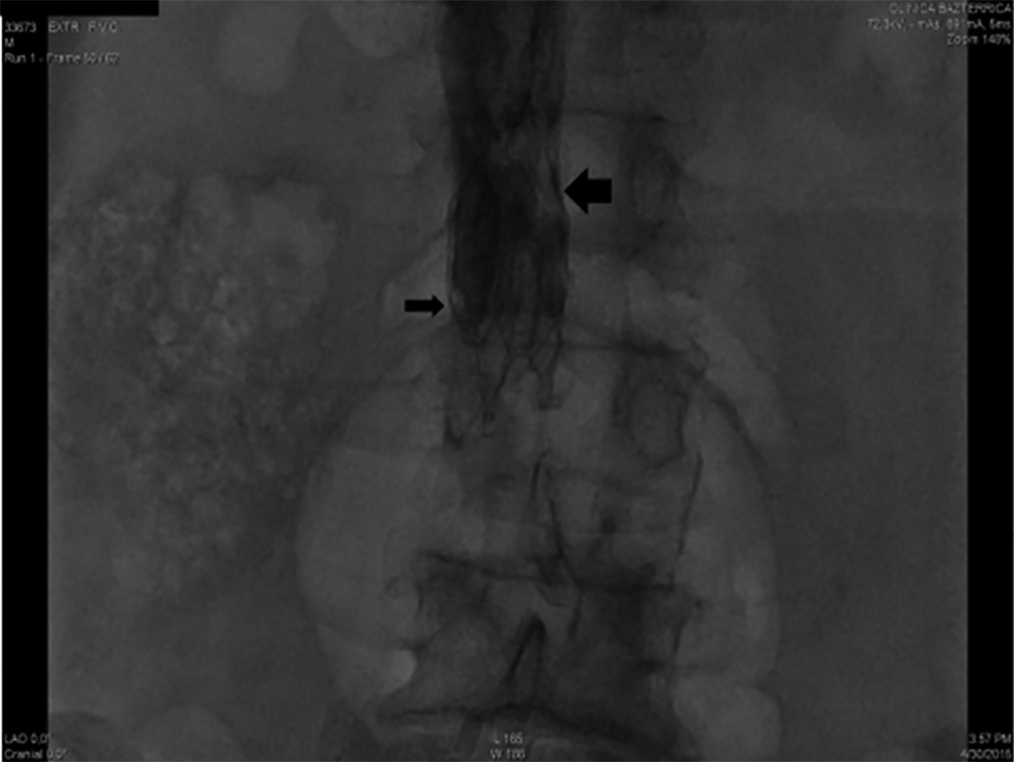
Inferior vena cava filter with trapped thrombi. The inferior vena cava filter was left in place after the extracorporeal membrane oxygenation run due to the trapped thrombi in the filter despite adequate anticoagulation treatment during extracorporeal membrane oxygenation.

Due to difficult weaning, the patient was tracheostomized on day 14. He required video-assisted thoracoscopy for left-side empyema due to *Klebsiella Pneumoniae* and was weaned from the ventilator on day 18 and decannulated on day 21. Partial right internal jugular vein thrombosis was diagnosed on follow-up, and anticoagulation was switched to oral acenocoumarol. The inferior vena cava filter was left in place due to evidence of thrombi trapped in it (Figure 3). After a short period on the general ward, he was discharged home without organ dysfunctions.

## DISCUSSION

In thoracic trauma patients, ECMO has the ability to artificially maintain cardiopulmonary function while the damaged organ recovers.^(3)^ It has been demonstrated to be a feasible and safe method in this particular population.^(4)^ Thrombotic complications are one of the reasons for the increase in morbidity and mortality after major trauma.^(5)^ However, in particular cases, anticoagulation is contraindicated due to bleeding in the traumatized areas. Our patient developed distal bilateral calf muscle vein thrombosis, and anticoagulation was indicated due to the high risk imposed by his condition.^(6)^ As previously described, an inferior vena cava filter was inserted due to progression of left-side hemothorax that forced the termination of anticoagulation. When the patient condition deteriorated and venovenous-ECMO was indicated, the following options were considered: to remove the inferior vena cava filter and use a femoro-jugular site cannulation with full anticoagulation, to use the femoro-jugular approach with the femoral cannula inserted proximal to the inferior vena cava filter with the tip below the renal veins or to use the DL cannula with no anticoagulation (at least the first 24 hours) while leaving the inferior vena cava filter in place. The strategy to leave the tip of the cannula below the renal veins has the potential to limit ECMO flow by inferior vena cava wall collapse due to suction,^(7)^ and this option was discarded. inferior vena cava filter extraction was considered but immediate full anticoagulation was deemed not to be safe due to previous hemothorax. Due to the risk of thrombus progression, if the femoral vein were to be cannulated, we decided to use a 27 French Avalon^®^. As was previously described, there was no interaction between the inferior vena cava filter and ECMO system, and the distance between the two intravascular devices remained unchanged. In our patient, anticoagulation had been associated with thoracic bleeding, and heparin was withheld during the beginning of the run, as has been previously reported.^(4)^

Gothner et al.^(8)^ described the use of the DL cannula in trauma patients, but none have been described to have a previously inserted inferior vena cava filter. Luk et al.^(9)^ described the insertion of an inferior vena cava filter prior to ECMO decannulation as a prophylactic approach to lower the risk of embolism. Femoro-jugular cannulation is traditionally preferred in ARDS patients due to higher flows permitted by the cannulas.^(7)^ However, Pappalardo et al.^(10)^ recently described no difference in blood flow or oxygenation with the use of the DL cannula compared with femoro-jugular cannulation. To the best of our knowledge, this is the first description of the need to use a DL cannula for venovenous-ECMO due to a previously inserted inferior vena cava filter.

## CONCLUSION

We described a case of thoracic trauma with severe acute respiratory distress syndrome patient who underwent venovenous extracorporeal membrane oxygenation via a double lumen right internal jugular with a previously inserted inferior vena cava. There were no complications related to inferior vena cava filter migration due to the extracorporeal membrane oxygenation system generated suction in the inferior vena cava. The use of the double lumen right internal jugular cannula could be a safe and feasible option in patients who cannot be cannulated via the femoral veins due to a previously inserted intravascular device.
